# The role of glutamate oxaloacetate transaminases in sulfite biosynthesis and H_2_S metabolism

**DOI:** 10.1016/j.redox.2020.101800

**Published:** 2020-11-24

**Authors:** Anna-Theresa Mellis, Albert L. Misko, Sita Arjune, Ye Liang, Katalin Erdélyi, Tamás Ditrói, Alexander T. Kaczmarek, Peter Nagy, Guenter Schwarz

**Affiliations:** a Institute for Biochemistry, Department of Chemistry, University of Cologne, Cologne, Germany; b Department of Neurology, Massachusetts General Hospital and Harvard Medical School, Boston, USA; c Department of Molecular Immunology and Toxicology, National Institute of Oncology, Budapest, Hungary; d Department of Anatomy and Histology, University of Veterinary Medicine, Budapest, Hungary; e Center for Molecular Medicine, University of Cologne, Cologne, Germany

**Keywords:** 6): cysteine catabolism, Sulfite oxidase, Glutamate oxaloacetate transaminase, H_2_S, Persulfidation, sulfide:quinone oxidoreductase

## Abstract

Molybdenum cofactor deficiency and isolated sulfite oxidase deficiency are two rare genetic disorders that are caused by impairment of the mitochondrial enzyme sulfite oxidase. Sulfite oxidase is catalyzing the terminal reaction of cellular cysteine catabolism, the oxidation of sulfite to sulfate. Absence of sulfite oxidase leads to the accumulation of sulfite, which has been identified as a cellular toxin. However, the molecular pathways leading to the production of sulfite are still not completely understood. In order to identify novel treatment options for both disorders, the understanding of cellular cysteine catabolism – and its alterations upon loss of sulfite oxidase – is of utmost importance. Here we applied a new detection method of sulfite in cellular extracts to dissect the contribution of cytosolic and mitochondrial glutamate oxaloacetate transaminase (GOT) in the transformation of cysteine sulfinic acid to sulfite and pyruvate. We found that the cytosolic isoform GOT1 is primarily responsible for the production of sulfite. Moreover, loss of sulfite oxidase activity results in the accumulation of sulfite, H_2_S and persulfidated cysteine and glutathione, which is consistent with an increase of SQR protein levels. Surprisingly, none of the known H_2_S-producing pathways were found to be upregulated under conditions of sulfite toxicity suggesting an alternative route of sulfite-induced shift from oxidative to H_2_S dependent cysteine catabolism.

## Highlights

• Cellular sulfite detection enables investigation of cysteine catabolism in cell extracts.
• Cytosolic glutamate oxaloacetate transaminase 1 is the primary source for cellular sulfite.
• Deficiency of sulfite oxidase leads to accumulation of H_2_S and persulfidated small molecules.
• Sulfite oxidase deficiency results in a downregulation of H_2_S synthesis and increased SQR expression.

## Graphical abstract

**Alterations in cysteine catabolism following sulfite oxidase (SO) deficiency.** Cysteine catabolism in wildtype and SO-deficient cells. Steps and enzymes that localize to mitochondria are depicted by the light blue background. The area outside refers to the cytosol. Cytosolic glutamate oxaloacetate transaminase 1 (GOT1) is responsible for cellular sulfite production, whereas mitochondrial GOT (GOT2), plays an important role in H_2_S production in SO deficiency. In absence of SO, sulfite (SO_3_^2−^) and hydrogen sulfide (H_2_S) accumulate. SO deficiency furthermore further impacts cellular cysteine catabolism resulting in lower intracellular cysteine levels and downregulation of many involved enzymes (depicted in gray letters). Moderate increase in GOT2 and strong induction of sulfide:quinone oxidoreductase (SQR) suggest an increased flux of sulfur via the GOT2-MPST-SQR axis. Abbreviations: CBS, cystathionine β-synthase; CSE, cystathionine γ-lyase; CDO, cysteine dioxygenase; GOT, glutamate oxaloacetate transaminase; MPST, mercaptopyruvate sulfurtransferase; SQR, sulfide:quinone oxidoreductase; ETHE1, persulfide dioxygenase; TST, thiosulfate sulfurtransferase.

**Figure:**
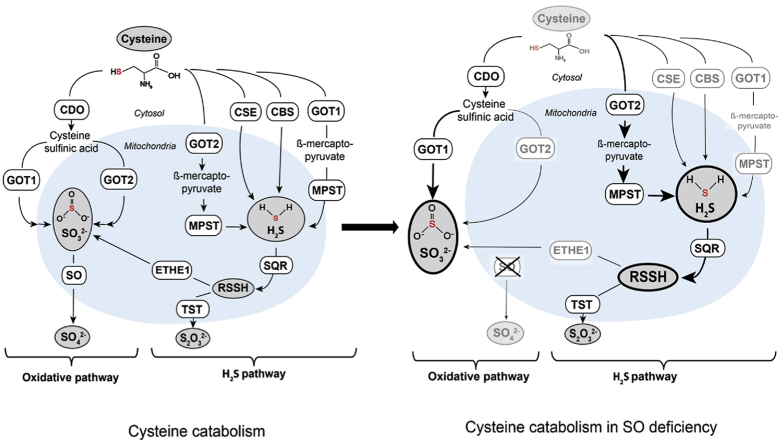

## Abbreviations

CARS2: cysteinyl-tRNA synthetase
CBS: Cystathionine β-synthase
CDO: Cysteine dioxygenase
CSA: Cysteine sulfinic acid
CSE: Cystathionine γ-lyase
CysSH: cysteine, persulfidated
EE: Ethylmalonic encephalopathy
ETHE1: Ethylmalonic encephalopathy protein 1
GOT: Glutamate oxaloacetate transaminase
GSH: Glutathione, reduced
GSSG: Glutathione, oxidized
GSSH: Glutathione, persulfidated
HPE-IAM: β-(4-hydroxyphenyl)ethyl iodoacetamide
ISOD: Isolated sulfite oxidase deficiency
LC-ESI-MS/MS: liquid chromatography-electrospray ionization-tandem mass spectrometry
MoCD: Molybdenum cofactor deficiency
SO: Sulfite oxidase
SQR: Sulfide:quinone oxidoreductase
SSC: S-sulfocysteine
TST: Thiosulfate sulfurtransferase

## 1 Introduction

Sulfite is a highly reactive molecule which is produced by the catabolism of the sulfur-containing amino acid cysteine, a semi-essential amino acid [1]. Cysteine is either taken up from dietary sources or enzymatically produced from methionine via the transsulfuration pathway [2]. Cysteine plays a pivotal role in protein structure due to its high reactivity and ability to form disulfides in an oxidative environment [3]. In addition, cysteine is also the key precursor for the biosynthesis of glutathione, which is the pivotal and highly abundant antioxidant peptide in the cell [4]. Furthermore, cysteine serves as a sulfur source for various types of cofactors, such as coenzyme A, biotin, Fe–S clusters and the molybdenum cofactor.

Non-protein bound cysteine is catabolized via two distinct processes, 1) the oxidative pathway and 2) various H_2_S-generating pathways. Both branches of cysteine catabolism converge in the production of sulfite and its detoxification by the mitochondrial molybdenum cofactor-containing enzyme sulfite oxidase (SO) [5]. H_2_S is an important signaling molecule in mammals that has gained increasing attention in recent years due to its versatile functions in cardio-vascular systems, neuronal tissues and in the gastrointestinal tract [6,7].

H_2_S can be produced via different enzymatic routes including 1) cystathionine β-synthase (CBS [8]), 2) cystathionine γ-lyase (CSE [9]) and 3) mercaptopyruvate sulfurtransferase (MPST [10]) in conjunction with glutamate oxaloacetate transaminase (GOT [11]). Intracellular H_2_S levels are tightly controlled by its biogenesis and efficient oxidative catabolism. The canonical H_2_S oxidation pathway proceeds through a series of mitochondrial enzymes ultimately yielding thiosulfate and sulfate.

The key enzyme in mitochondrial H_2_S oxidation is sulfide:quinone oxidoreductase (SQR), which, depending on its co-substrate, is able to produce S-sulfhydrylated (persulfidated) proteins or low molecular weight persulfides such as glutathione persulfide (GSSH) [12,13]. S-sulfhydrylation of cysteine residues in proteins has been shown to mediate a protective function on redox-active cysteine residues as well as to modulate enzymatic activities [[14], [15], [16]]. In addition to SQR, MPST has been reported to produce GSSH, CysSH and hydrogen polysulfides (H_2_S_n_) [17,18]. Recently, formation of cysteine persulfide (CysSH) by mitochondrial cysteinyl-tRNA synthetase (CARS2) has also been observed [[19], [20], [21]], suggesting a complex interplay of various enzymes in H_2_S-mediated modification of thiols. GSSH is oxidized by persulfide dioxygenase (ETHE1 [22]), yielding sulfite and GSH. Alternatively, persulfidated GSH may be converted to thiosulfate and GSH by thiosulfate sulfurtransferase (TST, also referred to as rhodanese) in a sulfite-dependent manner [23]. Persulfidated proteins however, have been reported to be primarily turned over by the thioredoxin system [24,25], which also results in H_2_S production.

During the oxidative catabolism, cysteine is first converted to cysteine sulfinic acid (CSA) via oxidation of its thiol group by the cytosolic enzyme cysteine dioxygenase (CDO). CSA is either decarboxylated to yield hypotaurine and taurine [26] or deaminated to release β-sulfinyl pyruvate, which spontaneously decomposes to pyruvate and sulfite [27]. CSA deamination was found to be catalyzed by the enzyme GOT [27], for which two isoforms are known, one localized in the cytosol (GOT1), the other in the mitochondrial matrix (GOT2) [28]. To this day, it is not known, which of the two isoforms is primarily responsible for the production of sulfite in a cellular context.

Cysteine catabolism via the oxidative pathway or the generation of H_2_S merge in the production of sulfite (via GOT1/2 or ETHE1, respectively), which is rapidly oxidized by SO. Deficiency of SO leads to a toxic accumulation of sulfite [29,30]. Cellular effects of sulfite toxicity include decreased intracellular NADH and ATP concentrations, depolarization of the mitochondrial membrane potential, impaired mitochondrial respiration, and inhibition of the enzymes glutamate- and malate dehydrogenase [[31], [32], [33]]. Moreover, sulfite reduces disulfide-bridges, thus affecting protein folding, stability and activity on a global scale. Sulfite-dependent cleavage of disulfide-bridges produces S-sulfonated species, such as the sulfite-cysteine adduct S-sulfocysteine (SSC) [34,35]. In contrast to this, it could recently be shown that, in physiological concentrations (1 μM), extracellular sulfite protects neurons from oxidative stress by positively influencing cellular GSH levels [36]. There are two known disorders that result from an inherited deficiency of SO activity, molybdenum cofactor deficiency (MoCD) and isolated SO deficiency (ISOD). Whereas ISOD is caused by mutations in the SO-encoding *SUOX* gene, MoCD arises from mutations in several genes, which are required for the synthesis of the molybdenum cofactor of SO [29]. MoCD and ISOD patients typically present in the neonatal period with similar symptoms including acute encephalopathy, intractable seizures, abnormal muscle tone, apnea, and feeding difficulties. Patients exhibit rapid neurological deterioration accompanied by panlobular cerebral atrophy [37], often leaving patients only with residual brainstem functions.

Despite significant progress that had been made with the development of a metabolic substitution therapy for MoCD [38], the underlying pathophysiology of MoCD and ISOD caused by sulfite toxicity remains poorly understood. In this respect, the intracellular sources of sulfite production and the impact of sulfite accumulation on cysteine catabolism needs to be uncovered.

In this study we investigated the role of cytosolic and mitochondrial GOT enzymes in oxidative and H_2_S-dependent cysteine catabolism. We identified cytosolic GOT1 as main sulfite-producing enzyme whereas mitochondrial GOT2 contributed to cellular H_2_S metabolism in SO deficiency. In addition, sulfite accumulation in SO deficiency was associated with increased H_2_S steady state levels and increased persulfidation of glutathione and cysteine providing a novel link between oxidative and H_2_S-dependent cysteine catabolism.

## 2 Material & methods

### 2.1 Cell culture conditions

Human embryonic kidney 293 cells with the insertion of Flp-In™ T-REx™ site (HEK293T) and all cell lines generated from these were kept at 37 °C, 5% CO_2_. The cells were cultivated in Dulbecco's Modified Eagle Medium (DMEM) (Pan Biotech), supplemented with 2 mM glutamine (gibco™ by Life Technologies) and 10% Fetal Bovine Serum (FBS) (Pan Biotech; Origin: South Africa). HEK293T *SUOX* ^−/−^ cells were originally described in Ref. [39].

### 2.2 Generation of cell lines via CRISPR/Cas9

*GOT1-*or *GOT2*-deficient HEK293T cells were generated in a *SUOX*-deficient background [39] using a modified protocol from Ref. [40]. In short, *GOT1* and *GOT2* specific Cas9 short guiding (sg)RNAs were predicted using Benchling software and selected for highest efficiency and lowest off-target rate (GOT1: CACCGCAGTCATCCGTGCGATATGC, GOT2: CACCGCCCCTCCTGCGCCGGCCTCA). Selected sgRNAs were inserted in a pX335 vector (Addgene #42335) and all constructs were transfected using Genejuice® (Merck Millipore) transfection reagent according to instructions. After 3 days, cells were isolated by limiting dilution and single colonies were grown in 96-well plates until further analysis. Knockout candidates were selected via Western Blot and confirmed by subsequent Sanger sequencing (Eurofins Genomics) using the following primers: (GOT1 forward; TCCTTCACTGTTCTGTTTTGACC), (GOT1 reversed; GGCAAGACGAGAAGCACAG; (GOT2 forward; CCCTGTCCTTACCTTCAGCA), (GOT2 reversed; TGCGTGTGAGTGACAGTGTG).

### 2.3 Sulfite detection

Sulfite was measured from cell extracts by a newly developed well plate-based assay using the sulfite-detecting dye CZBI [41]. Cells were grown to confluency on a 10 cm cell culture dish and resuspended in 500 μl sulfite extraction buffer (100 mM Tris/HCl pH 7.4; 100 mM Na_2_SO_4_, 0.05% deoxycholic acid). In order to break the cells, the lysate was frozen in liquid nitrogen and thawn at room temperature under vigorous vortexing. Afterwards, the cell suspension was centrifuged at 13000×*g* for 30 min at 4 °C to remove the cell debris. The supernatant was loaded onto a G25 column (No. 28918007, GE Healthcare) for de-proteinization. After loading, proteins were eluded in 1 ml extraction buffer and discarded. Next, the metabolites (including sulfite) were eluded in 1.5 ml extraction buffer and collected for further analysis in a 15 ml tube. In the meantime, a 1 mM CZBI stock solution was prepared using pure ETOH. This stock was further diluted to a 10 μM CZBI working solution in 3:7 ETOH/PBS with 1% BSA. For the measurement, 80 μl of the CZBI working solution were added per well (samples were measured in triplicates) in a black 96 well-plate. Next, 80 μl de-proteinized sample or extraction buffer as blank were added to the wells and mixed by swirling the plate. Since the CZBI dye is ratiometric, the fluorescence was detected at ex/em 322/480 and ex/em 510/580 in a platereader (Tecan Spark). For quantification, the intensity measured at ex/em 322/480 was divided by the one at ex/em 510/580 and then converted to the corresponding concentration with the help of a sulfite standard curve. Finally, all samples were normalized to their respective protein concentration (measured via Bradford assay).

### 2.4 Cell proliferation assay

Cell proliferation of the different cell lines was assessed by the MTT assay as described previously [34]. In brief, 1 × 10^4^ cells/well were seeded onto a 96-well and grown for 48 h. Afterwards, the old medium was exchanged with fresh phenol red-free DMEM containing 100 μM (3-(4,5-dimethylthiazol-2-yl)-2,5-diphyenyltetrazolium bromide (MTT). After a 4 h incubation at 37 °C in the dark, the MTT solution was exchanged for 100% DMSO. The plate was then placed in a shaker for 10–30 min at 37 °C until all cells were solubilized. Finally, the absorption was measured at 570 nm (reference 650 nm) in well plate reader (Tecan Spark).

### 2.5 Transfection of cells

For the overexpression of CDO, cells were transfected using polyethylenimine (PEI) [42]. Briefly, for transfection of a 10 cm^2^ dish, 1 ml DMEM without additives and 51.2 μl PEI (1 mg/ml, pH 6.8) were incubated for 5 min. After incubation, 12.8 μg of the respective plasmid was added and incubated for additional 20 min. The mixture was then added to the plate in small droplets. After 24 h, 5 ml fresh medium were added to each plate. Cells were ready for harvest and subsequent experiments after 48 h.

### 2.6 Determination of protein concentration

The concentration of proteins from cell extracts was determined by means of the Bradford assay. Therefore, 190 μl diluted Bradford solution (1/5 dilution) was incubated with 10 μl of protein solution (respective dilution) for 20 min and the absorption change at 595 nm was measured using a well plate reader (Biotek, Germany). The determined absorption change was then compared to that of standard proteins with known concentration in order to determine the protein concentration of unknown solutions.

### 2.7 Western Blot analysis

Western blotting was performed on crude protein extracts from HEK293T cells lysed in 100 mM Tris/Ac, pH 8.0. Protein concentration was adjusted accordingly after determination with the Bradford assay. Unless otherwise indicated, 30 μg of protein lysate were separated by SDS-PAGE and immunoblotted using a standard semi-dry blotting protocol onto PVDF membranes. The following antibodies were used to detect the respective proteins: Recombinant Anti-Sulfite oxidase antibody [EPR7618] (Abcam Cat# ab129094, RRID:AB_11143396); SQRDL Antibody (Novus Cat# NBP1-84510, RRID:AB_11027088); GOT2 Monoclonal Antibody, (3E9), (Thermo Fisher Scientific Cat# MA5-15595, RRID:AB_10981108); Recombinant Anti-ETHE1 antibody, [EPR11697], (Abcam, ab174302); CBS, (D8F2P), (Cell Signaling Technology Cat# 14782, RRID:AB_2798609); Recombinant Anti-TST antibody [EPR11645(B)], (Abcam Cat# ab166625, RRID:AB_2753194); Anti-GOT1 Antibody, clone 1F5.2 (Merck Millipore, MAB5508); MPST Antibody (Novus Cat# NBP1-82617, RRID:AB_11014969). β-tubulin was used as a loading control (Sigma-Aldrich Cat# T7816, RRID:AB_261770). The CSE-specific antibody was kindly provided by the laboratory of Solomon Snyder, University of Baltimore. Proteins were then visualized using HRP-coupled anti-mouse (Santa Cruz Biotechnology Cat# sc-2055, RRID:AB_631738) and anti-rabbit (Santa Cruz Biotechnology Cat# sc-2054, RRID:AB_631748) secondary antibodies. The signals were detected using chemiluminescent substrates (ThermoFisher Scientific, #34580) and a BioRad ChemiDoc XRS + system.

For densitometric measurements, images were converted to 8-bit format using ImageJ (NIH, version 1.50i). Lanes were selected using the square tool and the histograms for individual bands were quantified after background removal. Identical squares were used for each quantified band. Unless otherwise stated, signals were normalized to β-tubulin as loading control and expressed relative to WT levels.

### 2.8 GOT activity assay

The activity assay for recombinant expressed human GOT isoforms was conducted for aspartate and CSA in a total volume of 100 μl containing the following components: 100 mM MES buffer pH 8.0, 20 mM α-KG, 20 mM PLP, 0.015 μM GOT isoforms and varying concentrations of substrates (0–20 mM). The reaction was started by addition of substrate and incubated at 37 °C with vigorous shaking to ensure proper oxygenation using a thermomixer (Eppendorf). The GOT-catalyzed reaction was terminated by the addition of 100 μl 5% sulfosalicylic acid and centrifugation for removal of precipitated proteins followed by quantification of the reaction product glutamate via HPLC. For the determination of total GOT activity from cell extracts, a commercial Aspartate Aminotransferase (AST or SGOT) Activity Colorimetric Assay Kit (BioVision) was used following the instructions of the manufacturer.

### 2.9 HPLC measurement

For detection of glutamate generated during the kinetic measurement of both GOT isoforms, derivatization using the fluorescent dye *o*-phthalaldehyde (OPA) was used. This method is based on the HPLC –based analysis of SSC [43]. Preparation of the derivatization reagent was based on the protocol of Jakoby and Griffith [44] and achieved by dissolving 1 g of OPA in 10 ml methanol, mixed with 90 ml 0.4 M borate buffer pH 10.2 and 400 μl 2-mercaptoethanol. Pre-column derivatization was carried out using an automated autosampler, which was programmed to mix 25 μl of the sample with 5 μl of derivatization reagent. After an incubation time of 0.2 min, the mixture was injected and the injection valve was bypassed to achieve the derivatization of the next sample, thus eliminating the time needed for the derivatization procedure. Detection was carried out by fluorescence (excitation 240 nm, emission 450 nm). HPLC analyses were carried out using an Agilent 1100 SL system (Agilent Technologies GmbH, Böblingen, Germany) consisting of a binary pump (SL series), vacuum degasser, autosampler, thermostated column compartment (SL series), diode array detector (SL series), fluorescence detector, all controlled by Agilent ChemStation software (Version Rev. B.04.01. SP1 (647)). The mobile phase consisted of buffer A (10 mM Na2HPO4; 10 mM Na2B4O7) and eluent B (45% acetonitrile 45% methanol and 10% water). Separation was achieved using an XBridge column (75 × 4.6 mm, 2.5 μm, Waters GmbH, Eschborn, Germany) and isocratic elution with 7% eluent B.

For calculation of kinetic parameters, the initial reaction velocity was determined with the slope obtained from the area determined via HPLC analysis for one time point per reaction. Peak areas were plotted against time and linear regression for the initial phase was determined corresponding to the initial rate. At least five time points of the kinetic curve were used for fitting to calculate the reaction rate at a given substrate concentration. After subtracting the velocity of the negative controls, the reaction mix without substrate, the substrate-dependent velocities v were used for determining the kinetic parameter Km and vmax.

### 2.10 LC-MS/MS analyses of persulfide and sulfide species

For the analysis of intracellular persulfide levels, β-(4-Hydroxyphenyl) ethyl iodoacetamide (HPE-IAM) labeling was used based on a previous publication [19]. The method was carried out as described here: 4 × 10^5^ HEK293T cells were seeded in 12 well plates and cultured overnight. Cultured cells were washed twice with PBS and collected in ice-cold 75% methanol solution containing 5 mM HPE-IAM (Santa Cruz Biotechnology), sonicated and incubated at 37 °C for 20 min. After centrifugation at 14000*g* for 10 min at 4 °C, aliquots of the supernatants were acidified with formic acid (5% final) and diluted with 0.1% formic acid containing known amounts of isotope-labeled internal standards for LC-MS/MS analysis. Cell pellets were dissolved in 1% SDS/PBS and protein contents were measured using BCA assay. An Orbitrap Q Exactive Focus mass spectrometer (Thermo Scientific) coupled to a Vanquish UHPLC (Thermo Scientific) system was used to perform LC-ESI-MS/MS analysis. Per/polysulfide derivatives were separated on a Kinetex C18 column (Phenomenex, 50 × 2.0 mm, 2.6 μm) under the following elution conditions: mobile phases A (0.1% formic acid in water) and B (0.1% formic acid in methanol) were used to produce a linear gradient from 5% B to 90% B in 15 min at a flow rate of 0.3 ml/min at 30 °C. MS/MS spectra were obtained in positive ionization mode with an ESI source at 1,5 KV and 250 °C, fragmentation was achieved with higher-energy collisional dissociation (HCD). Various per/polysulfide derivatives were identified and quantified by means of single reaction monitoring (SRM). The *m*/*z* values of parent and fragment ions can be found in Table 1.

**Table 1:** MS/MS Detection parameters for persulfide analysis.

Analyte	Parent *m*/*z*	Fragment *m*/*z*	Collision energy (eV)
Cys	299.1	121.1	34
Cys (int. std)	300.1	121.1	34
Cys-persulfide	331.1	121.1	35
Cys-persulfide (int. std)	333.1	121.1	35
GSH	485.1	356.1	16
GSH-persulfide	517.1	388.1	18
H_2_S	389.1	121.1	38

## 3 Results

### 3.1 Both GOT isoforms use CSA as a substrate *in vitro*

The two GOT isoenzymes (GOT1 and GOT2) catalyze a wide variety of reversible transamination reactions [11,27,28]. Initially, they were found to facilitate the reaction of aspartate with α-ketoglutarate yielding glutamate and oxaloacetate. Based on the structural similarity of CSA to the primary GOT substrate aspartate, the enzyme has been suggested to catalyze the deamination of CSA resulting in the formation of β-sulfinyl pyruvate and glutamate [27,28]. Since β-sulfinyl pyruvate is unstable, it spontaneously decomposes into sulfite and pyruvate, thus making GOT the final enzymatic step in the oxidative catabolism of cysteine preceding sulfite production [27].

**Fig. 1:**
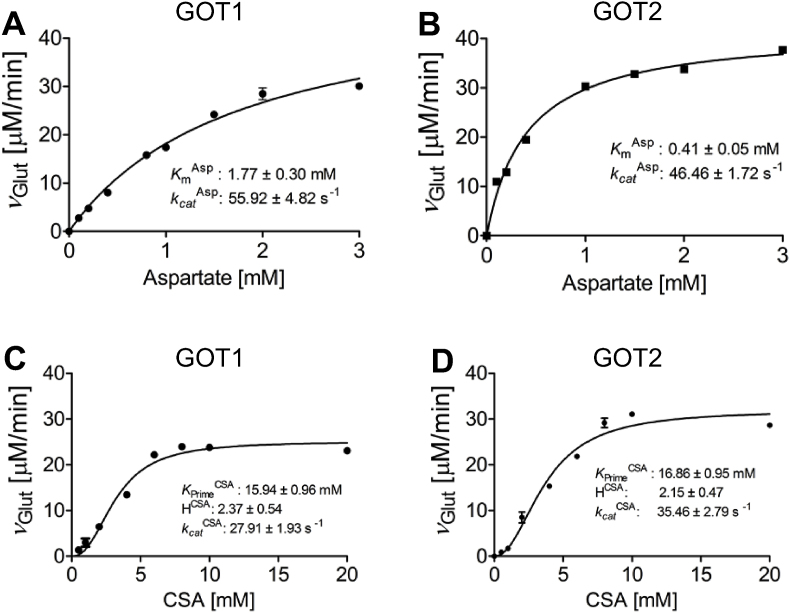
**Kinetic characterization of human GOT1 and GOT2 isoenzymes**. **A./B.** Steady state kinetics of human GOT1 (A) and GOT2 (B) using aspartate as substrate. Activity of purified GOT (1.5 μM) was determined using standard assay conditions and HPLC detection of the reaction product glutamate. Error bars indicate standard deviations (n = 3). **C./D.** Steady state kinetics of human GOT1 (C) and GOT2 (D) using cysteine sulfinic acid (CSA) as substrate. Activity of purified GOT (1.5 μM) was determined using standard assay conditions and HPLC detection of the reaction product glutamate. Error bars indicate standard deviations (n = 3).

To probe whether both GOT isoforms are able to catalyze the deamination of CSA, we conducted kinetic measurements at 37 °C using either CSA or aspartate as canonical substrate with α-ketoglutarate and monitored the formation of glutamate via HPLC. Results revealed striking differences between the affinities towards the different substrates, while the turnover of the respective isoforms was comparable (Fig. 1A–D). Aspartate as a substrate revealed a typical Michaelis-Menten type of reaction for both GOT isoforms (Fig. 1B). In light of the similar *k*_cat_ values of 46–56 s^−1^, one can conclude that the lower *K*_*m*_^Asp^ of 0.41 mM for GOT2 is indicative of a higher affinity for aspartate as compared to the cytosolic GOT1 isoform for which a *K*_*m*_^Asp^ of 1.77 mM was determined.

In contrast, steady state kinetics using CSA as a substrate showed a sigmoidal curve, pointing to a positive cooperativity within both GOT isoforms upon deamination of CSA (Fig. 1C and D). *K*_Prime_^CSA^ of 15.9 mM and 16.9 mM were determined for the cytosolic GOT1 and mitochondrial GOT2 isoforms, respectively. Comparison of the catalytic efficiencies of both GOT isoforms clearly support aspartate as the primary substrate as an approximately 28-fold and 50-fold increase in catalytic efficiency for aspartate against CSA was determined for GOT1 and GOT2, respectively. In addition to earlier studies [27,28], we confirm CSA-dependent formation of glutamate, which further supports a possible role of both GOT isoforms in the production of sulfite during cysteine catabolism.

### 3.2 GOT1 is the main producer of intracellular sulfite

**Fig. 2:**
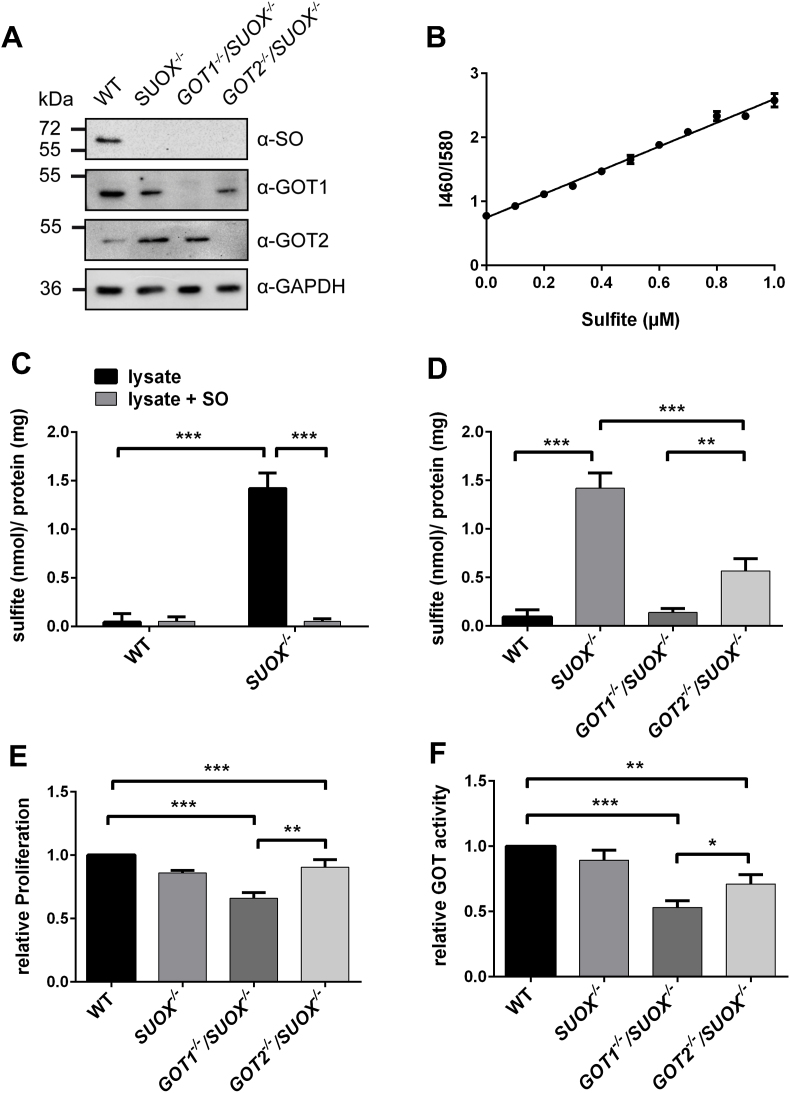
**Contribution of GOT1 and GOT2 to cellular sulfite production**. **A.** Western Blot of HEK293 lysates confirming knockout of SO, GOT1 and GOT2 (n = 3). **B.** Sulfite standard curve used for the determination of sulfite concentration from cellular extracts (n = 3). **C.** Sulfite measurement from cell extracts of WT and *SUOX*^*−/−*^ cells with and without addition of purified SO (n = 4). Sulfite levels were normalized to cellular protein concentration. Error bars indicate standard deviation. Two-way ANOVA with Tukey's post-hoc test for pairwise comparisons was performed as indicated. *p* value: *** <0.001; ** <0.01; * <0.05; ns > 0.05. **D.** Sulfite measurement from cell extracts of WT, *SUOX*^*−/−*^, *GOT1*^*−/−*^/*SUOX*^*−/−*^ and *GOT2*^*−/−*^/*SUOX*^*−/−*^ cells (n = 4). Sulfite levels were normalized to cellular protein concentration. Error bars indicate standard deviation. One-way ANOVA with Tukey's post-hoc test for pairwise comparisons was performed as indicated. *p* value: *** <0.001; ** <0.01; * <0.05; ns > 0.05. **E.** Relative (isoform-unspecific) GOT activity determined from extracts of WT, *SUOX*^*−/−*^, *GOT1*^*−/−*^/*SUOX*^*−/−*^ and *GOT2*^*−/−*^/*SUOX*^*−/−*^ cells (n = 3). Values were adjusted to cellular protein concentration and normalized to WT. Error bars indicate standard deviation. One-way ANOVA with Tukey's post-hoc test for pairwise comparisons was performed as indicated. *p*value: *** <0.001; ** <0.01; * <0.05; ns > 0.05. **F.** Relative cell proliferation of WT, *SUOX*^*−/−*^, *GOT1*^*−/−*^/*SUOX*^*−/−*^ and *GOT2*^*−/−*^/*SUOX*^*−/−*^ cells determined by MTT assay (n = 3). Values were adjusted to cellular protein concentration and normalized to WT. Error bars indicate standard deviation. One-way ANOVA with Tukey's post-hoc test for pairwise comparisons was performed as indicated. *p* value: *** <0.001; ** <0.01; * <0.05; ns > 0.05.

To investigate, which of the two GOT isoforms is primarily responsible for sulfite production in cells, we generated *GOT1*^−/−^/*SUOX*^–/–^ and *GOT2*^−/−^/*SUOX*^–/–^ double knockout (KO) cells via CRISPR/Cas9, by disrupting the *GOT1* or *GOT2* genes in a *SUOX*-deficient HEK293 background (Fig. 2A). While *SUOX*-deficient cells are expected to accumulate sulfite, deletion of either of the two GOT isoforms should suppress sulfite accumulation. To quantify sulfite in cellular extracts, we applied the fluorescent ratiometric sensor CZBI [45] in a modified buffer system that enhanced the sensitivity of the sulfite detection. The amount of sulfite was quantified by comparison with a standard curve (Fig. 2B).

We found that *SUOX*^–/–^ cells accumulated on average 1.4 nmol/mg protein sulfite, which was approximately 15 times more than detected in WT HEK293T cells (Fig. 2C). This accumulation is a direct consequence of SO loss of function, as the addition of purified SO to *SUOX*^–/–^ cell lysate reduced the sulfite amount to WT levels. Next, we quantified cellular sulfite levels from *GOT1*^−/−^/*SUOX*^–/–^ and *GOT2*^−/−^/*SUOX*^–/–^ double knockout (KO) cells (Fig. 2D). Strikingly, in the absence of the cytosolic GOT1 isoform, intracellular sulfite accumulation caused by SO deficiency was abolished. Loss of the mitochondrial GOT2 isoform also led to a significant decrease in sulfite levels compared to *SUOX*^*−/−*^ cells; however, measured sulfite concentrations were still about 5 times higher than those in *GOT1*^−/−^/*SUOX*^–/–^ cells. It should be noted, that there was no sulfite accumulation in *GOT1*^*−/−*^ or *GOT2*^*−/−*^ cells (Fig. S1). Additionally, we isolated mitochondria from the respective cell lines and measured sulfite accumulation from cytosolic and mitochondrial fractions (Fig. S2). In all three KO cell lines, sulfite was only found in the cytosolic fraction and could not be detected within mitochondria. Furthermore, we determined total GOT activity from cellular extracts and found a significant reduction in *GOT1*^−/−^/*SUOX*^–/–^ cells to 50% of the WT, while the *GOT2*^−/−^/*SUOX*^–/–^ cells retained 70% of WT activity (Fig. 2E). These data show that while the presence of either GOT isoform is sufficient to maintain basic GOT activity, the overall contribution of GOT1 appears to be greater. Cellular growth was also more impaired in *GOT1*^−/−^/*SUOX*^–/–^ cells than *GOT2*^−/−^/*SUOX*^–/–^ cells, highlighting again the importance of GOT1 (Fig. 2F). In summary, our data collectively point towards GOT1, the cytosolic isoform, as the main producer of sulfite in cells.

### 3.3 Sulfite production depends on cellular cysteine- and CDO levels

**Fig. 3:**
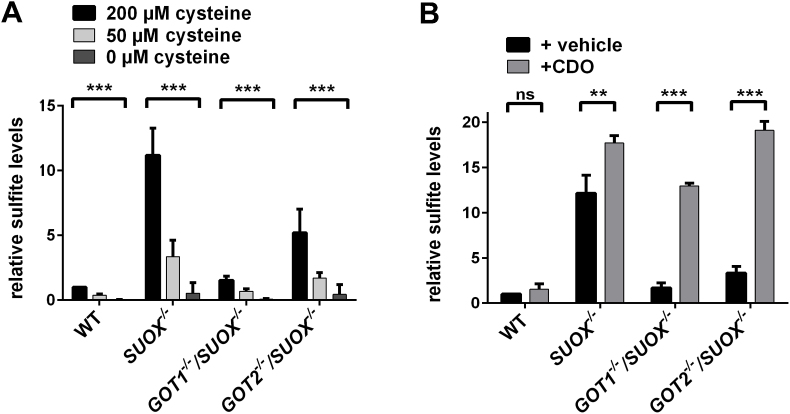
**Dependence of sulfite production on cellular cysteine- and CDO levels**. **A.** Sulfite measurement from cell extracts of WT, *SUOX*^*−/−*^, *GOT1*^*−/−*^/*SUOX*^*−/−*^ and *GOT2*^*−/−*^/*SUOX*^*−/−*^ cells after incubation in different cysteine concentrations (n = 4). Sulfite levels were adjusted to cellular protein concentration. Values were normalized to WT. Error bars indicate standard deviation. Two-way ANOVA with Tukey's post-hoc test for pairwise comparisons was performed as indicated. *p* value: *** <0.001; ** <0.01; * <0.05; ns > 0.05. **B.** Sulfite measurement from cell extracts of WT, *SUOX*^*−/−*^, *GOT1*^*−/−*^/*SUOX*^*−/−*^ and *GOT2*^*−/−*^/*SUOX*^*−/−*^ cells following overexpression of CDO (n = 4). Sulfite levels were adjusted to cellular protein concentration. Values were normalized to WT. Error bars indicate standard deviation. Two-way ANOVA with Tukey's post-hoc test for pairwise comparisons was performed as indicated. *p* value: *** <0.001; ** <0.01; * <0.05; ns > 0.05.

It is assumed that cysteine catabolism is the sole source of intracellular sulfite [1]. We therefore measured sulfite production following cysteine deprivation in order to assess for other possible sources of sulfite formation. We incubated WT, *SUOX*^–/–^, *GOT1*^−/−^/*SUOX*^–/–^ and *GOT2*^−/−^/*SUOX*^–/–^ cell lines in cysteine- and cystine-free media where we added either 200 μM cysteine (initial concentration of cysteine in DMEM), 50 μM cysteine, or no cysteine (Fig. 3A). In all cell types, including the WT, we found a clear decrease in cellular sulfite production following reduction or deprivation of cysteine. Under cysteine deprivation, the sulfite production of the *SUOX*^–/–^ or either double KO cell line was reduced to WT level or even lower. These data clearly demonstrate the dependence of cellular sulfite production on cysteine, confirming that cellular cysteine catabolism is the major, if not the only source of sulfite production.

CDO, the first enzyme in the oxidative pathway of cysteine catabolism is often referred to as the “master regulator” of cysteine catabolism [2]. Upon increased cysteine availability, CDO concentrations in hepatic and adipose tissue increase by up to 45‐fold [46]. In contrast, in the absence of cysteine, CDO levels were found to be decreased due to ubiquitination and subsequent proteasomal degradation of the enzyme. We thus tested the contribution of CDO in the production of sulfite by overexpressing CDO in all four cell lines (Fig. S3) and subsequently measured the corresponding sulfite levels in those cells (Fig. 3B). CDO overexpression increased sulfite levels in all cell lines except for WT. While there was only a modest increase in the *SUOX*^–/–^ cells, the effect was much more pronounced in the double knockout cells, with *GOT2*^−/−^/*SUOX*^–/–^ cells showing the highest accumulation. This finding is in agreement with our previous findings that GOT1 is the major enzyme in sulfite production. These data indicate that CDO, along with cysteine, plays an important role in the cellular production of sulfite.

### 3.4 Persulfidation- and H_2_S levels are elevated in *SUOX*^–/–^ cells

**Fig. 4:**
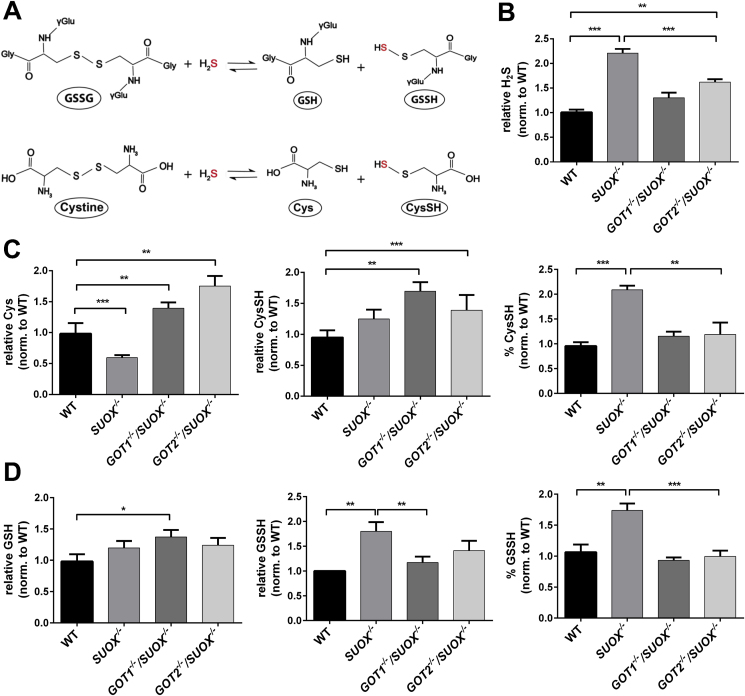
**Determination of cellular levels of H**_**2**_**S, persulfidated cysteine and –glutathione in SUOX**^**−/−**^**cells**. **A.** Putative production of persulfidated cysteine (CysSH) and –glutathione (GSSH). **B.** Determination of H_2_S levels from WT, *SUOX*^*−/−*^, *GOT1*^*−/−*^/*SUOX*^*−/−*^ and *GOT2*^*−/−*^/*SUOX*^*−/−*^ cells. Error bars indicate standard deviation. One-way ANOVA with Tukey's post-hoc test for pairwise comparisons was performed as indicated. *p* value: *** <0.001; ** <0.01; * <0.05; ns > 0.05. **C.** Determination of total free cysteine, free cysteine persulfide and the percentage of persulfidated cysteine from WT, *SUOX*^*−/−*^, *GOT1*^*−/−*^/*SUOX*^*−/−*^ and *GOT2*^*−/−*^/*SUOX*^*−/−*^ cells. Error bars indicate standard deviation. One-way ANOVA with Tukey's post-hoc test for pairwise comparisons was performed as indicated. *p* value: *** <0.001; ** <0.01; * <0.05; ns > 0.05. **D.** Determination of total glutathione, glutathione persulfide and the percentage of persulfidated glutathione from WT, *SUOX*^*−/−*^, *GOT1*^*−/−*^/*SUOX*^*−/−*^ and *GOT2*^*−/−*^/*SUOX*^*−/−*^ cells. Error bars indicate standard deviation. One-way ANOVA with Tukey's post-hoc test for pairwise comparisons was performed as indicated. *p* value: *** <0.001; ** <0.01; * <0.05; ns > 0.05.

We next wanted to test whether deficiency of SO or GOT1/2 would also impact the other branch of cysteine catabolism, the H_2_S metabolic pathways. It is important to note that GOT enzymes also function as cysteine oxaloacetate transaminases [11,47,48], yielding 2-mercaptopyruvate, which is further desulfurated by MPST and releases H_2_S. Levels of free H_2_S have been examined in multiple studies and – depending on the employed method and investigated tissue – were found in concentrations ranging from 144.5 nmol/g in mouse liver [49] to 3 μM in mouse brain [50]. In addition, H_2_S levels were linked to persulfides of small molecules such as cysteine (CysSH) and glutathione (GSSH). We therefore measured levels of free H_2_S in comparison to cysteine- and glutathione (GSH) persulfidation levels (Fig. 4A) using liquid chromatography-electrospray ionization-tandem mass spectrometry (LC-ESI-MS/MS) with β-(4-hydroxyphenyl)ethyl iodoacetamide (HPE-IAM) as a trapping agent according to Ref. [19]. Due to its mild electrophilicity, HPE-IAM ensures specific labeling of persulfides and minimizes its artificial, alkylation induced decay [51].

We found that H_2_S levels were increased in all KO cell lines compared to WT (Fig. 4B). *SUOX*^–/–^ cells displayed the highest amount of H_2_S with a more than two-fold increase compared to WT, whereas the *GOT1*^−/−^/*SUOX*^–/–^ cells only displayed a mild increase (~1.2 fold) The *GOT2*^−/−^/*SUOX*^–/–^ cells displayed approx.1.5 times more H_2_S than WT cells. These data indicate that loss of SO results in a strong accumulation of H_2_S while concomitant loss of GOT1 or GOT2 partially rescues the observed H_2_S increase. Again, loss of GOT2 activity was less suppressive than loss of GOT1.

Next, we investigated cysteine persulfidation (CysSH) and found that total cellular levels of free cysteine were diminished in *SUOX*^–/–^ cells, while the amount of CysSH was similar to WT cells, thus leading to a significant increase in the percentage of CysSH in *SUOX*^–/–^ cells (Fig. 4C). In contrast, both double KO cell lines displayed increased free cysteine levels compared to WT cells. However, the amount of CysSH was also increased in the double KO cells lines, which translated into an equal percentage of CysSH in both double KO cell lines compared to WT. Furthermore, the *SUOX*^–/–^ cells displayed higher total amounts of persulfidated GSH (GSSH) compared to WT cells, as well as a higher percentage of GSSH (Fig. 4D). Although we measured slightly higher total GSH levels in the *GOT1*^−/−^/*SUOX*^–/–^ cells compared to WT, GSSH percentage and total amount in either double KO cell line were equal to WT levels. Therefore, it appears that loss of either cytosolic GOT1 or mitochondrial GOT2 is sufficient to lower the increased persulfidation levels observed in *SUOX*^–/–^ cells back down to WT levels.

### 3.5 Altered protein expression levels suggest a redirected catabolic flux via the H_2_S pathway

In order to further understand potential alterations caused by sulfite accumulation on H_2_S metabolism, we performed WB analysis of the proteins known to be involved in H_2_S oxidation and generation.

**Fig. 5:**
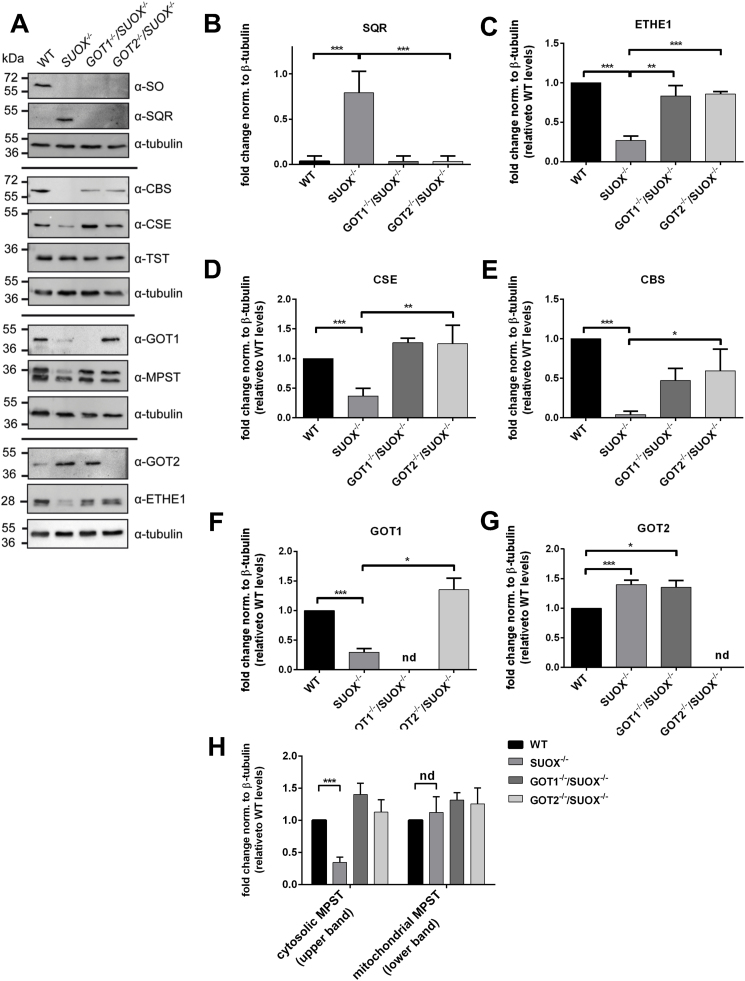
**Expression levels of proteins involved in H**_**2**_**S metabolism**. **A.** Expression levels of proteins involved in cysteine catabolism (SO, SQR, CBS, CSE, TST, GOT1, MPST, GOT2 and ETHE1) in WT, *SUOX*^*−/−*^, *GOT1*^*−/−*^/*SUOX*^*−/−*^ and *GOT2*^*−/−*^/*SUOX*^*−/−*^ cells. Tubulin was used as a loading control. **B.** Quantification of expression changes of SQR in relation to tubulin and WT samples (n = 4). Error bars indicate standard deviation. One-way ANOVA with Tukey's post-hoc test for pairwise comparisons was performed as indicated. *p* value: *** <0.001; ** <0.01; * <0.05; ns > 0.05.**C.** Quantification of expression changes of ETHE1 in relation to tubulin and WT samples (n = 4). Error bars indicate standard deviation. One-way ANOVA with Tukey's post-hoc test for pairwise comparisons was performed as indicated. *p* value: *** <0.001; ** <0.01; * <0.05; ns > 0.05. **D.** Quantification of expression changes of CSE in relation to tubulin and WT samples (n = 4). Error bars indicate standard deviation. One-way ANOVA with Tukey's post-hoc test for pairwise comparisons was performed as indicated. *p* value: *** <0.001; ** <0.01; * <0.05; ns > 0.05. **E.** Quantification of expression changes of CBS in relation to tubulin and WT samples (n = 4). Error bars indicate standard deviation. One-way ANOVA with Tukey's post-hoc test for pairwise comparisons was performed as indicated. *p* value: *** <0.001; ** <0.01; * <0.05; ns > 0.05. **F.** Quantification of expression changes of GOT1 in relation to tubulin and WT samples (n = 4). Error bars indicate standard deviation. One-way ANOVA with Tukey's post-hoc test for pairwise comparisons was performed as indicated. *p* value: *** <0.001; ** <0.01; * <0.05; ns > 0.05. **G.** Quantification of expression changes of GOT2 in relation to tubulin and WT samples (n = 4). Error bars indicate standard deviation. One-way ANOVA with Tukey's post-hoc test for pairwise comparisons was performed as indicated. *p* value: *** <0.001; ** <0.01; * <0.05; ns > 0.05. **H.** Quantification of expression changes of MPST (cytosolic and mitochondrial isoforms) in relation to tubulin and WT samples (n = 4). Error bars indicate standard deviation. One-way ANOVA with Tukey's post-hoc test for pairwise comparisons was performed as indicated. *p* value: *** <0.001; ** <0.01; * <0.05; ns > 0.05.

First, we found that SQR, the main mediator of H_2_S oxidation and a producer of cellular persulfides, was drastically (more than 7-fold) upregulated in *SUOX*^−/−^ cells (Fig. 5A and B). This finding agrees with the observed increase of SQR products such as persulfidated glutathione and cysteine. In contrast, *GOT1*^−/−^/*SUOX*^−/−^ or *GOT2*^−/−^/*SUOX*^−/−^ cells, which showed WT-like persulfidation levels of GSH or cysteine, showed again SQR protein levels comparable to WT. In line with increased GSSH, ETHE1, which catalyzes the conversion of GSSH to sulfite and GSH, was in turn downregulated in *SUOX*^–/–^ cells, but not in *GOT1*^−/−^/*SUOX*^–/–^ or *GOT2*^−/−^/*SUOX*^–/–^ cells (Fig. 5A and B). Similarly, *GOT1*^*−/−*^ and *GOT2*^*−/−*^ single KO cells displayed protein expression patterns comparable to WT (Fig. S4). The other enzyme involved in H_2_S oxidation, TST, did not appear to be altered in any KO cell line (Fig. 5A) suggesting a minor role of thiosulfate production in HEK293 cells. This result is consistent with our finding that only low and similar levels of thiosulfate were detected in all four cell lines (data not shown).

Next we asked, which of the different biosynthetic pathways might have contributed to the elevation of H_2_S in *SUOX*^–/–^ cells. Surprisingly, CSE and CBS, the reportedly predominant enzymes of cellular H_2_S synthesis [52] were both significantly downregulated in *SUOX*^–/–^ cells. In contrast, CSE levels in *GOT1*^−/−^/*SUOX*^–/–^ and *GOT2*^−/−^/*SUOX*^–/–^ cells were comparable to WT and CBS levels partially restored to WT levels (Fig. 5A, C). The third path towards H_2_S involves GOT and MPST in either compartment, cytosol and mitochondria. Unexpectedly, GOT1 protein levels were severely reduced in *SUOX*^–/–^ cells, whereas GOT2 protein levels were increased (Fig. 5F and G). While the later remained unchanged in *GOT1*^−/−^/*SUOX*^–/–^ cells, the lack in GOT1 expression was normalized in *GOT2*^−/−^/*SUOX*^–/–^ cells, suggesting that both isoforms can at least partially compensate for each other. Finally and consistently, expression of cytosolic MPST was found to be strongly reduced in *SUOX*^–/–^ cells and again rescued in both double KO cell lines, while mitochondrial MPST showed no significant change in expression in any of the cells.

**Fig. 6:**
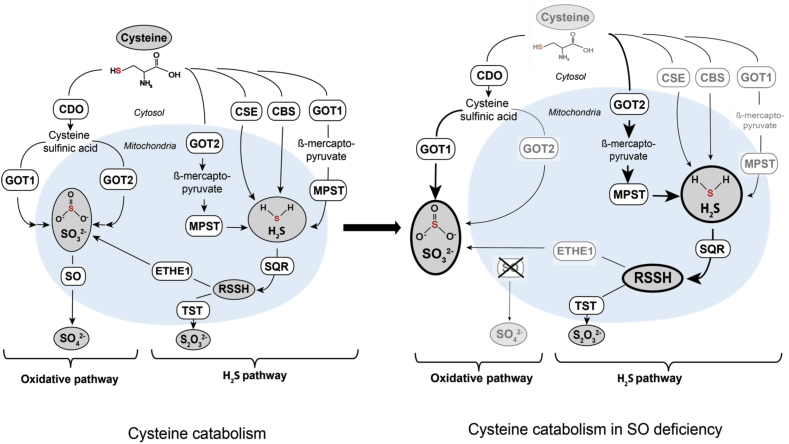
Alterations in cysteine catabolism followingsulfite oxidase (SO) deficiency. Cysteine catabolism in WT and SO-deficient cells. Steps and enzymes that localize to mitochondria are depicted by the light blue background. The area outside refers to the cytosol. Cytosolic glutamate oxaloacetate transaminase 1 (GOT1) is responsible for cellular sulfite production, whereas mitochondrial GOT (GOT2), plays an important role in H_2_S production in SO deficiency. In absence of SO, sulfite (SO_3_^2−^) and hydrogen sulfide (H_2_S) accumulate. SO deficiency furthermore further impacts cellular cysteine catabolism resulting in lower intracellular cysteine levels and downregulation of many involved enzymes (depicted in gray letters). Moderate increase in GOT2 and strong induction of sulfide:quinone oxidoreductase (SQR) suggest an increased flux of sulfur via the GOT2-MPST-SQR axis. Abbreviations: CBS, cystathionine β-synthase; CSE, cystathionine γ-lyase; CDO, cysteine dioxygenase; GOT, glutamate oxaloacetate transaminase; MPST, mercaptopyruvate sulfurtransferase; SQR, sulfide:quinone oxidoreductase; ETHE1, persulfide dioxygenase; TST, thiosulfate sulfurtransferase.

In summary, we show that protein levels in all three H_2_S-producing pathways were diminished in *SUOX*^–/–^ cells, thereby suggesting a suppressive function of accumulating sulfite (Fig. 6). On the other hand, increased levels of H_2_S correlated well with elevated SQR levels, more persulfidated cysteine and glutathione and less ETHE1 expression suggesting a higher flux of sulfur through the H_2_S pathway in SO deficiency.

## 4 Discussion

MoCD and ISOD are rare genetic disorders that lead to rapid and devastating neurodegeneration early in life. Both disorders result from the inactivation of SO and the subsequent accumulation of sulfite in the setting of unchecked production in patient tissues [30]. However, the enzyme that produces the majority of sulfite still remains elusive. Here we measured for the first time the sulfite accumulation in HEK293T based cell culture models of SO deficiency by applying an enhanced sulfite detection method based on a selective fluorescent sensor [41]. Furthermore, we performed functional and expression studies of key enzymes of cysteine catabolism. As a result, we show that the cytosolic enzyme GOT1 is the primary sulfite-producing enzyme, while the contribution of GOT2 appears to be rather restricted to its H_2_S-dependent role in sulfite formation.

Accumulation of thiosulfate, a product of H_2_S catabolism, has also been observed in MoCD and ISOD patients [1]. This suggests that loss of SO and its accompanying sulfite accumulation could also influence H_2_S metabolism. Thus, we investigated alterations in the overall flux of cysteine catabolism by quantifying the levels of free H_2_S and persulfidated small molecules. We found that H_2_S levels as well as persulfidated cysteine and GSH levels were highly elevated in *SUOX*^–/–^ cells, indicating a dysregulation of H_2_S metabolism. These accumulations were reversed upon knockout of GOT1 or GOT2, suggesting that GOT1 or GOT2 may also impact the homeostasis of the H_2_S pathway. However, future studies should address if this effect is directly mediated by the enzymes, or an indirect consequence of sulfite accumulation.

Both isoenzymes GOT1 and GOT2 are involved in the transamination of a wide variety of substrates in amino acid metabolism and thereby regulate a number of cellular processes besides cysteine catabolism [28,53]. Among those are the generation of intra-mitochondrial NADH by the malate-aspartate shuttle, which influences cellular redox balance and mitochondrial energy production via the electron transport chain [54,55]. Recently, the cytosolic isoform GOT1 has gained increasing attention for its role in promoting cancer cell proliferation by influencing cellular glutamate/glutamine metabolism [[56], [57], [58], [59], [60]]. Furthermore, GOT1 has been shown to be essential under conditions of mitochondrial dysfunction by providing cells with aspartate [61,62].

The reaction mechanism of the two GOT enzymes involves the PLP-dependent and reversible transfer of an amino group from an α-amino acid substrate to an α-keto acid. Here we show by direct detection of the co-product glutamate that human GOT1 and GOT2 catalyze the deamination of CSA *in vitro.* Since *k*_*cat*_ values are comparable for either isoform as well as both substrates (CSA and aspartate), reaction rates can be considered as similar. However, the difference in *K*_*M*_ values suggests that the preferred reaction is likely determined by the respective substrate concentration. While aspartate is highly abundant [63], intracellular concentrations of CSA could not be determined [2], which would suggest lower levels. Our data therefore confirm that deamination of aspartate would be favored under physiological conditions, which is in line with the numerous reports regarding the importance of aspartate synthesis and metabolism [56,62,64,65].

While our kinetic data did not show significant differences between the two GOT isoforms, we found that deletion of GOT1 was sufficient to revert sulfite accumulation in *SUOX*^–/–^ cells. *GOT1*^*−/−*^*/SUOX*^–/–^ cells were also more severely impaired in their growth and overall GOT activity. This finding adds to the accumulating evidence that cytosolic GOT1 is more important for overall cellular health than mitochondrial GOT2 [56,61,66]. However, we also measured a reduction in sulfite levels upon the deletion of GOT2 in *SUOX*^–/–^ cells, which would suggest that GOT2 might also impact cellular cysteine catabolism. Furthermore, our Western Blot studies showed that both GOT1 and GOT2 are reciprocally upregulated in the absence of the other isoform, suggesting that they can at least partially compensate for each other. However, it remains unclear for which of the different cellular functions of the GOT proteins this might be the case. In contrast to GOT1, recent reports of GOT2-deficiency in humans indicate that the most important cellular function of GOT2 appears to be related to the malate-aspartate shuttle [67]. Interestingly, mutations in *GOT2* were associated with early-onset of encephalopathy and epilepsy [67], which are also hallmarks of MoCD and ISOD pathophysiology.

Another disease that directly affects cellular cysteine catabolism is ethylmalonic encephalopathy or ETHE1 deficiency. Similarly to MoCD and ISOD, ETHE1 deficiency has also been associated with early-onset of progressive neurodegeneration and childhood death of patients [68]. Instead of sulfite, however, high levels of H_2_S and thiosulfate have been observed in body fluids and tissues of ETHE1 deficient patients [22,69]. On the molecular level, elevated levels of H_2_S have been reported to interfere with cellular respiration via inhibition of cytochrome *c* oxidase in muscle and brain [70]. As thiosulfate levels have also been found to be elevated in patients of MoCD or ISOD, we decided to further investigate H_2_S metabolism in *SUOX*^–/–^ cells. Indeed, we discovered that the levels of free H_2_S in SO deficient cells were more than two times higher than in WT cells, indicating a rewiring of cellular H_2_S metabolism. In line with this finding, ETHE1 protein levels were reduced in *SUOX*^–/–^ cells. However, in comparison to the reported 20-fold increase of H_2_S in a mouse model of ETHE1 deficiency [70], the observed two-fold increase in *SUOX*^–/–^ cells appears rather mild. It is therefore possible that the elevated H_2_S levels in SO deficiency might not be toxic, but instead exert protective effects. In fact, the important signaling function of H_2_S in vasorelaxation, inflammation, cell survival and cellular functions has been widely recognized in the past years [71]. It is furthermore becoming increasingly clear that the protective function of H_2_S is tightly linked to its ability to modify cysteine residues of proteins and small molecules in form of a post-translational modification called persulfidation [16,72,73]. Indeed, we found elevated levels of persulfidated GSH and cysteine in *SUOX*^–/–^ cells, along with highly elevated protein levels of SQR, the main mediator of GSH persulfidation. Decreased SQR protein levels have been reported in the mitochondrial disorder Coenzyme Q deficiency, which is also associated with increased H_2_S levels [74,75]. The drastic upregulation of SQR in *SUOX*^–/–^ cells might therefore protect against the accumulation of toxic amounts of H_2_S. Nevertheless, the underlying mechanism of how sulfite and/or H_2_S may control SQR expression remains to be elucidated.

In the past, the biogenesis of H_2_S has mostly been associated with the proteins CBS and CSE [52]. However, since KO mouse models of either CBS or CSE showed both WT-like H_2_S production in the brain, MPST has been proposed as an important producer of H_2_S in neuronal tissues [76,77]. Surprisingly, we found both CBS and CSE protein levels to be reduced in *SUOX*^–/–^ cells, as well as GOT1 and the cytosolic form of MPST. That would leave the GOT2-MPST pathway as the most likely remaining route for the observed increase in H_2_S production in *SUOX*^–/–^ cells. However, H_2_S levels could also be influenced by CARS2-dependent cysteine-persulfide formation [51]. Interestingly, while H_2_S oxidation is known to take place in the mitochondrial matrix, the GOT2-MPST axis is to date the only known intra-mitochondrial pathway generating free H_2_S. Indeed, GOT2 levels were highly increased in *SUOX*^–/–^ cells. Based on their kinetic properties in comparison to MPST, the GOT1/2 enzymes have been proposed to be the “gatekeepers” of this pathway [52]. The elevated GOT2 levels should therefore suffice to generate higher H_2_S levels via the GOT2-MPST axis, even though the levels of mitochondrial MPST are not altered. Both, CBS and CSE also play important roles in the transsulfuration pathway, the conversion of methionine to cysteine [2]. Their reduced protein levels might therefore be part of a feedback mechanism to reduce the overall flux through cysteine catabolism, especially the oxidative part, and thereby prevent excess sulfite production under conditions of SO deficiency.

## 5 Conclusions

The severity of disorders that are driven by alterations in cysteine catabolism highlight the importance of understanding the underlying connections between the oxidative and H_2_S dependent pathways that both converge in sulfite formation. Therefore, it remains to be determined how much sulfite is produced via ETHE1 under normal and pathological conditions. Generation of a CDO/SO double knockout model would likely answer this question. In aggregate, in this study we clarified that the main pathway of oxidative cysteine catabolism takes place in the cytosol and involves the GOT1 enzyme. Under conditions of SO deficiency, accumulating sulfite causes an increase in H_2_S and persulfidated species, which involves mitochondrial GOT2-dependent H_2_S biogenesis.

## Author contributions

A.-T.M. and G.S. conceptualized the study; A.L.M. and Y.L. developed the sulfite measurement, S.A. performed initial studies and recombinant GOT activity measurement; K.E. and D.T. performed H_2_S and persulfidation measurements under supervision of P·N.; A.T.K. provided *SUOX*^–/–^ cells; A.-T.M. and G.S. wrote the manuscript, which was reviewed by P.N., T.D., A.T.K, and A.L.M.

## Funding

This work was supported by the 10.13039/501100001659German Research Foundation (Deutsche Forschungsgemeinschaft, DFG) SFB1218 project number 269925409 and SFB1403 project number 414786233 the 2019 Hungarian Thematic Excellence Program (TUDFO/51757/2019-ITM) to P·N., by the Hungarian National Research, Development and Innovation Office KH_126766 and K_129286] to P.N. and the NINDS of the 10.13039/100000002National Institutes of Health (1K12NS098482-01) to A.L.M.

## Declaration of competing interest

The authors declare no competing interests. A.L.M. consults and receives research funding from Origin Biosciences and has submitted a provisonal patent application on sulfite detection methods. GS is CEO of Colbourne Pharmaceuticals and consults Origin Bioscience in the development of cPMP therapy.
