# Supplementary material for: The role of glutamate oxaloacetate transaminases in sulfite biosynthesis and H2S metabolism
Source: Redox Biol. 2020 Nov 24;38:101800. doi: 10.1016/j.redox.2020.101800 (PMC7711302; doi:10.1016/j.redox.2020.101800)
Supplement: Multimedia component 1 [file mmc1.pdf]

Supplementary figures

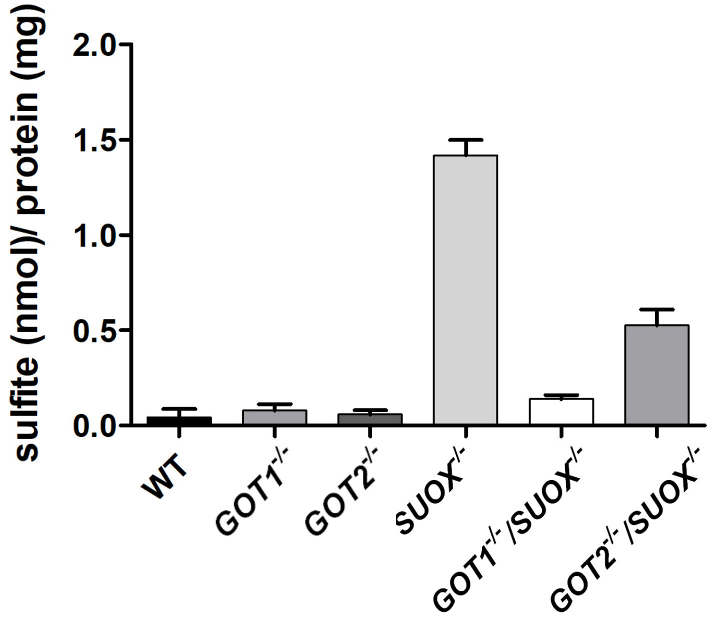

Fig. S1: Sulfite detection from *GOT1*$^{-/-}$ and *GOT2*$^{-/-}$ cells.
Sulfite was measured from WT, *SUOX*$^{-/-}$, *GOT1*$^{-/-}$, *GOT2*$^{-/-}$, *GOT1*$^{-/-}$/*SUOX*$^{-/-}$ and *GOT2*$^{-/-}$/*SUOX*$^{-/-}$ cell extracts (n = 3). Values were adjusted to cellular protein concentration and normalized to WT. Error bars indicate standard deviation.

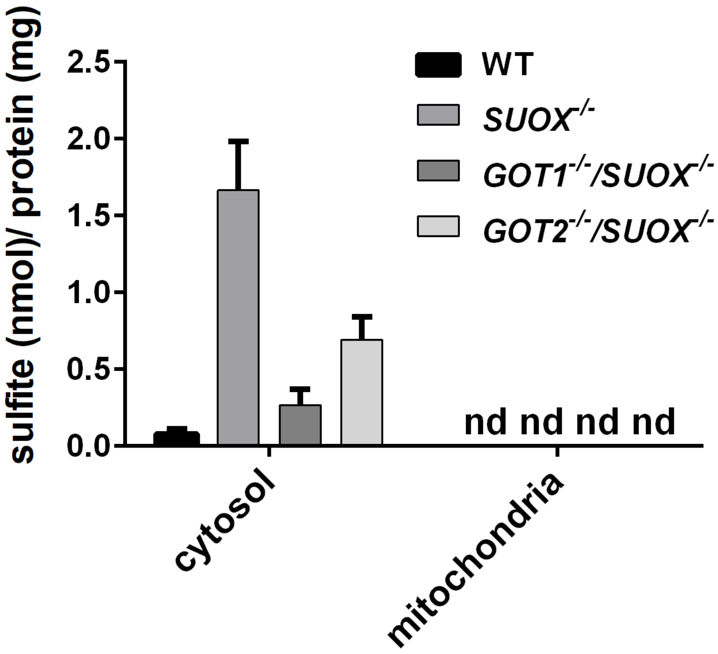

Fig. S2: Sulfite detection from mitochondrial fractions

Cytosolic and mitochondrial fractions from WT, *SUOX*$^{-/-}$, *GOT1*$^{-/-}$/*SUOX*$^{-/-}$ and *GOT2*$^{-/-}$/*SUOX*$^{-/-}$ cells were separated by differential centrifugation. Mitochondrial fractions were solubilized in sulfite assay buffer and tested for sulfite content (n = 3). Values were adjusted to cellular protein concentration and normalized to WT. Error bars indicate standard deviation.

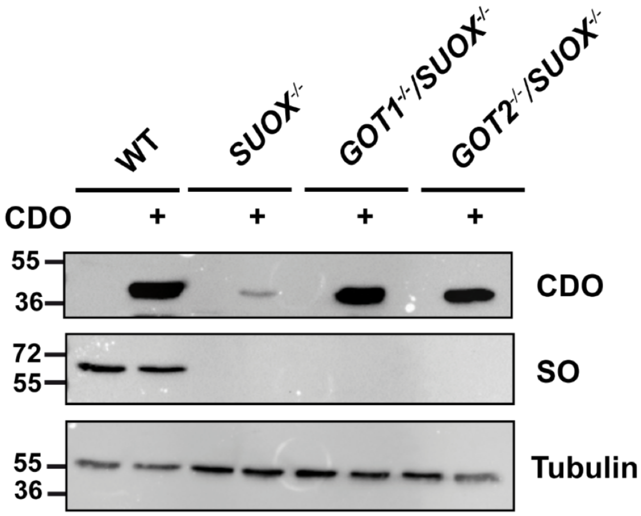

Fig. S3: CDO expression levels.
After transient transfection´of WT, *SUOX*$^{-/-}$, *GOT1*$^{-/-}$/*SUOX*$^{-/-}$ and *GOT2*$^{-/-}$/*SUOX*$^{-/-}$ cells with CDO, CDO expression levels were analyzed by WB in the respective cells lines (n = 3).

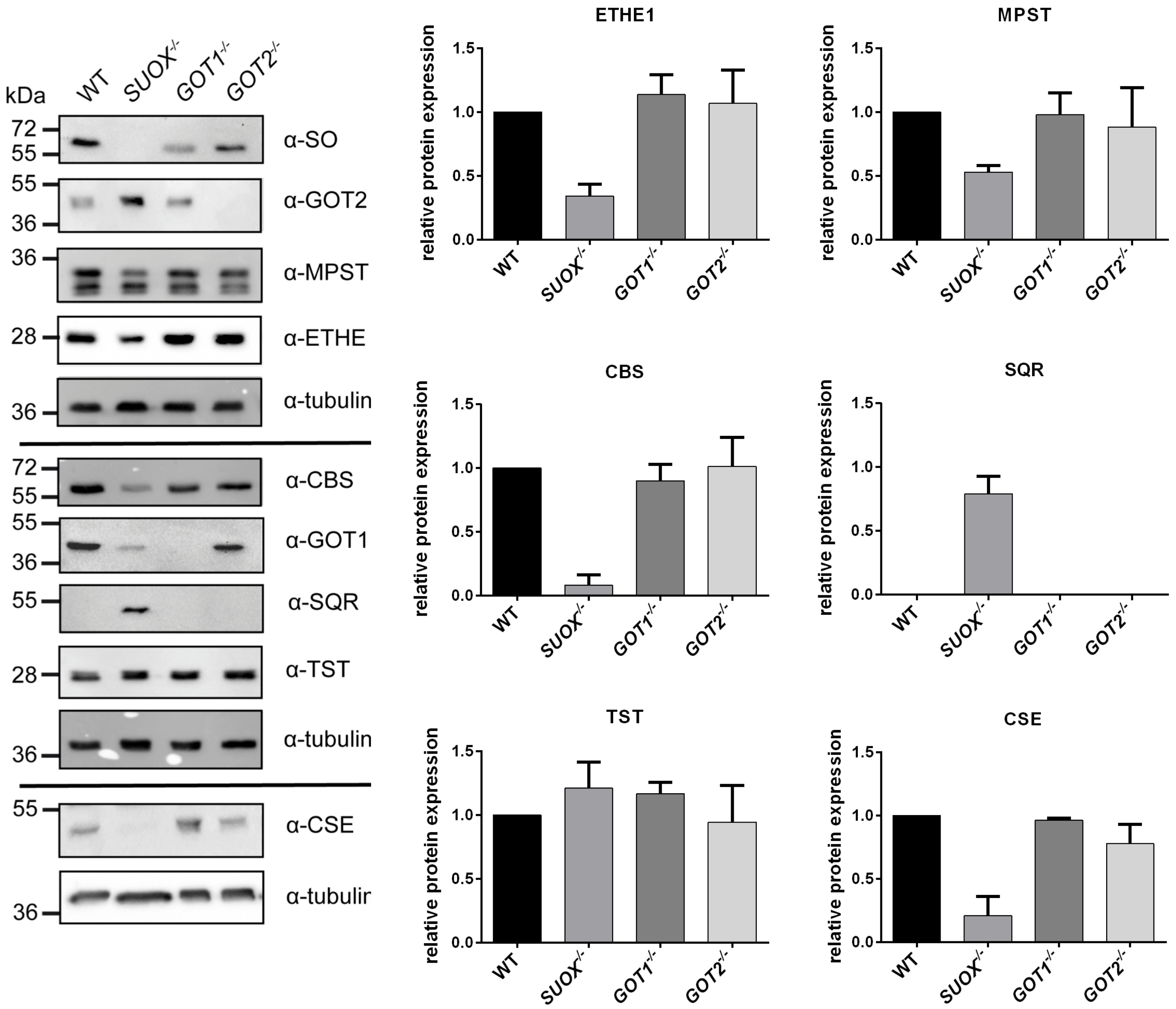

Fig. S4: Protein expression in *GOT1*$^{-/-}$ and *GOT2*$^{-/-}$ cells. Expression levels of proteins involved in cysteine catabolism (SO, SQR, CBS, CSE, TST, GOT1, MPST, GOT2 and ETHE1) in WT, *SUOX*$^{-/-}$, *GOT1*$^{-/-}$ *,* *GOT2*$^{-/-}$ *GOT1*$^{-/-}$/*SUOX*$^{-/-}$ and *GOT2*$^{-/-}$/*SUOX*$^{-/-}$ cells. Tubulin was used as a loading control. Quantification of expression changes is visualized in relation to tubulin and WT samples (n = 2).
